# New reference genomes to distinguish the sympatric malaria parasites, *Plasmodium ovale curtisi* and *Plasmodium ovale wallikeri*

**DOI:** 10.1038/s41598-024-54382-5

**Published:** 2024-02-15

**Authors:** Matthew Higgins, Emilia Manko, Daniel Ward, Jody E. Phelan, Debbie Nolder, Colin J. Sutherland, Taane G. Clark, Susana Campino

**Affiliations:** 1 Department of Infection Biology, Faculty of Infectious and Tropical Diseases, London School of Hygiene and Tropical Medicine, Keppel Street, London, WC1E 7HT UK; 2 UK Health Security Agency, Malaria Reference Laboratory, London School of Hygiene and Tropical Medicine, Keppel Street, London, WC1E 7HT UK; 3 Faculty of Epidemiology and Population Health, London School of Hygiene and Tropical Medicine, London, WC1E 7HT UK

**Keywords:** Computational biology and bioinformatics, Genome informatics

## Abstract

Despite *Plasmodium ovale curtisi* (*Poc*) and *wallikeri* (*Pow*) being important human-infecting malaria parasites that are widespread across Africa and Asia, little is known about their genome diversity. Morphologically identical, *Poc* and *Pow* are indistinguishable and commonly misidentified*.* Recent rises in the incidence of *Poc*/*Pow* infections have renewed efforts to address fundamental knowledge gaps in their biology, and to develop diagnostic tools to understand their epidemiological dynamics and malaria burden. A major roadblock has been the incompleteness of available reference assemblies (PocGH01, PowCR01; ~ 33.5 Mbp). Here, we applied multiple sequencing platforms and advanced bioinformatics tools to generate new reference genomes, Poc221 (South Sudan; 36.0 Mbp) and Pow222 (Nigeria; 34.3 Mbp), with improved nuclear genome contiguity (> 4.2 Mbp), annotation and completeness (> 99% *Plasmodium* spp., single copy orthologs). Subsequent sequencing of 6 *Poc* and 15 *Pow* isolates from Africa revealed a total of 22,517 and 43,855 high-quality core genome SNPs, respectively. Genome-wide levels of nucleotide diversity were determined to be 2.98 × 10^–4^ (*Poc*) and 3.43 × 10^–4^ (*Pow*), comparable to estimates for other *Plasmodium* species. Overall, the new reference genomes provide a robust foundation for dissecting the biology of *Poc/Pow*, their population structure and evolution, and will contribute to uncovering the recombination barrier separating these species.

## Introduction

*Plasmodium ovale curtisi* (*Poc*) and *Plasmodium ovale wallikeri* (*Pow*) are the least-studied human-infecting *Plasmodium* parasites. Large gaps remain in our understanding of these elusive parasites, from their full geographic distribution to antimalarial susceptibility^1,2^. Historically, *P. ovale* was considered a single parasite species (defined by blood-film morphology) associated with benign malaria which rarely presented severe complications including jaundice, anaemia, and fatal pulmonary impairments^3–5^. In 2010, it was demonstrated that ovale malaria was in fact caused by two non-recombining sympatric species subsequently named *Poc* and *Pow*^2^. *P. ovale* spp. infections mostly occur in Africa (94.5%) followed by Asia (5.3%)^6–10^. However, the prevalence of *P. ovale* spp*.* has been historically underestimated due to asymptomatic infections being unnoticed or undetected, a lack of an accurate rapid diagnostic test (RDT), and microscopy-based misclassification^11–15^. As such *P. ovale* spp*.* infections are commonly reported as mixed infections, alongside *P. falciparum* or *P. vivax*, where the presence of another parasite causes an infected individual to become symptomatic and seek medical attention^16–18^. Worryingly, a multi-centre study in Kenya reported an increase in the prevalence of *P. ovale* spp. co-infections between years 2008 and 2016^19^, but there is a lack of data from other settings. Such reports were prior to the SARS-CoV-2 pandemic, which has subsequently caused a global increase in malaria incidence across all *Plasmodium* species due to the disruption of intervention efforts^20,21^.

To date, the genomic characterization of *Poc* and *Pow* remains limited, especially when compared to other human infecting *Plasmodium* species such as *P. falciparum* and *P. vivax*^22^. Addressing this gap in our knowledge is vital, from the development of new treatments and diagnostics to facilitating accurate parasite population surveillance. Most genomic analyses require a complete reference genome, but both currently available assemblies (PocGH01 ~ 33.4 Mbp and PowCR01 ~ 33.5 Mbp; from Ghana) are incomplete, averaging > 2700 and > 7700 unknown nucleotides per megabase, respectively^23^. To fill this gap, we have sequenced two DNA samples (Poc221 and Pow222), sourced from mono-infections arising in Africa, utilising both Illumina and Oxford Nanopore Technology platforms. The resulting bioinformatic and validation analysis, provides two new reference genomes (Poc221 and Pow222), which are closer to being complete and more robust than the existing *P. ovale* spp. assemblies, and can be used for high resolution population genomic analysis across large numbers of sequenced samples.

## Results

### Assembly of the new reference genomes

For the generation of new *Poc* and *Pow* reference genomes, two DNA samples (Poc221, Pow222) from travellers returning to the UK from South Sudan and Nigeria respectively, were obtained from the UK Health Security Agency Malaria Reference Laboratory (UKHSA MRL). Both samples were initially sequenced using the Oxford Nanopore Technology (ONT) MinION platform under adaptive sampling conditions, negatively selecting against the human genome followed by *P. ovale* spp. specific enrichment via selective whole genome amplification (SWGA). The samples were subsequently sequenced using additional ONT MinION runs and the Illumina platform (see Methods). Together this yielded 13.68 Gb and 13.78 Gb of sequence data for samples Poc221 (11.58 Gb Illumina, 2.10 Gb ONT) and Pow222 (10.32 Gb Illumina, 3.46 Gb ONT), respectively. Following quality control (see Methods), 2.59 and 2.40 Gb of WGS data remained for samples Poc221 and Pow222, respectively (Supplementary Table S1). A hybrid assembly approach using Spades software was then implemented to generate the new reference sequences for Poc221 and Pow222 (see Supplementary Figure S1; Methods), which resulted in nuclear (14 chromosomes) and organellar (mitochondrion, apicoplast) genomes.

### Benchmarking to previous reference genomes

**Figure 1:**
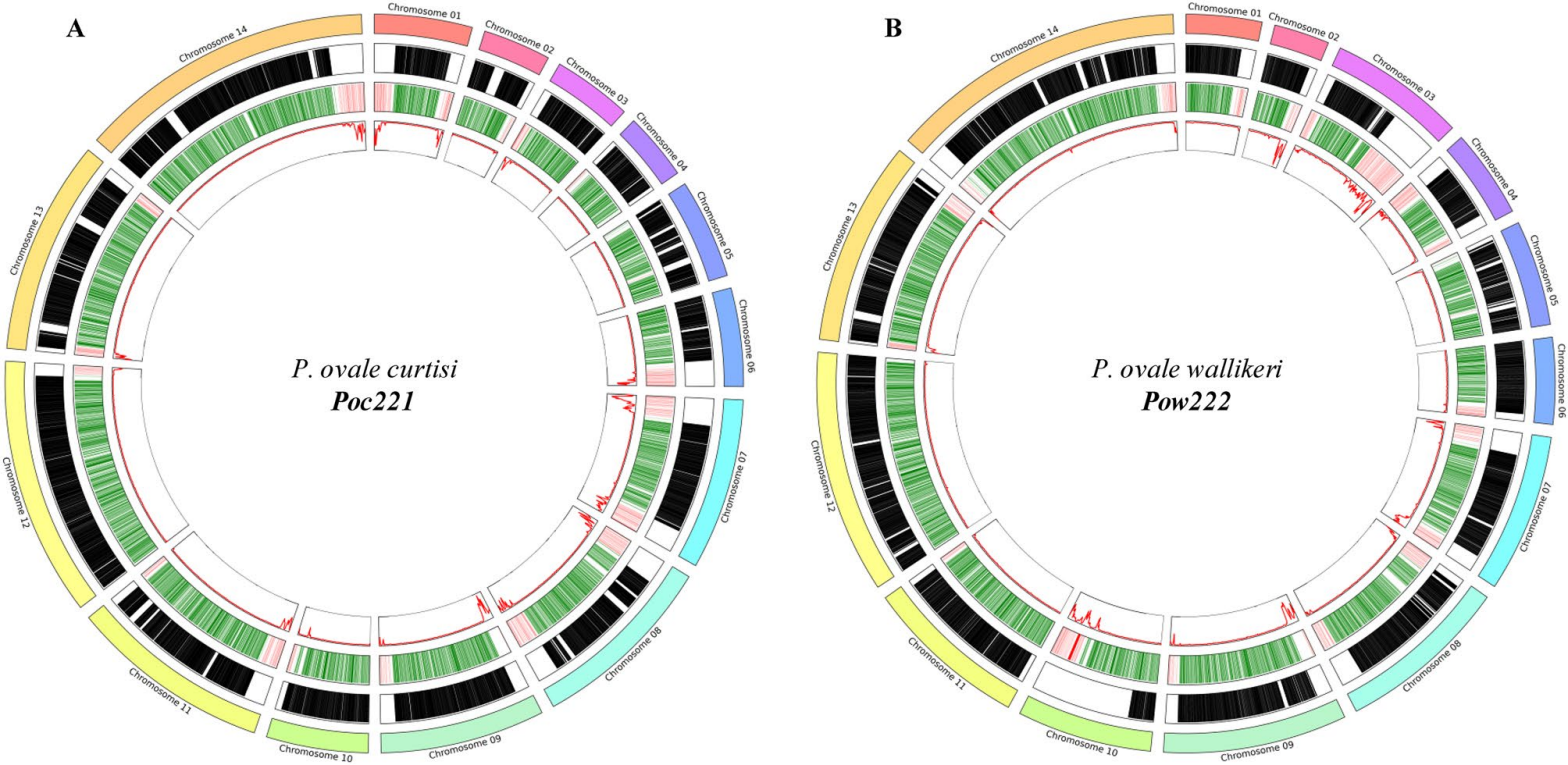
Visualizing the new genomic references (**A**) Poc221 and (**B**) Pow222. From outer ring to inside: (1) Representation of each nuclear chromosome; (2) Black regions represent chromosomal specific islands of homology identified between the new and historic references. (3). Green regions represent core ortholog genes identified in the new references and Red regions represent members of the PIR multigene family. 4) SNP density.

The new reference genomes were benchmarked against the existing available references, PocGH01 (https://plasmodb.org/: v54) and PowCR01 (https://www.ncbi.nlm.nih.gov/: GCA_900090025.2) (Table 1). Gains in core genome contiguity were made across both *P. ovale* ssp. (> 4.2 Mbp), reflected by > 5- and eightfold improvements in N50, a common metric to assess assembly quality, alongside a 76% and 86% reduction in the number of gaps for both *Poc* and *Pow*, respectively. When assessing contiguity improvements on a chromosomal level (Supplementary Figure S2), gains were made for 13 *Poc* chromosomes, with a maximum increase of at least 670 Kbp in chromosome 8 and minimum increase of at least 70 Kbp in chromosome 9. Similarly, gains were made across 13 *Pow* chromosomes, with a maximum increase of > 1.1 Mbp for chromosome 10. For both species, most contiguity gains were in sub-telomeric regions (Fig. 1). However, for *Pow* the most important contiguity gain was on chromosome 10, covering extended core and sub-telomeric regions, which were missing in the PowCR01 assembly. The *Poc* and *Pow* nuclear chromosomes have an average homology of 84.1% and 81.0%, respectively, between the new genomes and historic assemblies, which is again reflected in the comparable GC content obtained between new and historic references for each species (Table 1).

**Table 1:** Comparison of assembly metrics between available references for *P. ovale curtisi* and *P. ovale wallikeri.*

Species	ID (Source)	Scaffold count	Assembly size (Mbp)	Core Genome (Mbp)	GC content	Gap count per Mbp	N count per Mbp	N50
*P. ovale curtisi* *(Poc)*	Poc221 (S. Sudan)	1177	36.0	24.9	28.08	6	703	217,310
	PocGH01 (Ghana)	654	33.4	20.7	28.48	26	2713	38,586
*P. ovale wallikeri* *(Pow)*	Pow222 (Nigeria)	787	34.3	25.8	29.10	5	710	269,642
	PowCR01 (Ghana)	779	33.5	20.9	29.02	36	7778	30,524

### Annotation enhancements

Poc221 and Pow222 reference genomes were annotated using a combination of Companion and Metaeuk software (see Methods), and then compared to PocGH01 and PowCR01 (Table 2). The new reference genomes had an increased number of protein coding genes (*Poc* + 769, *Pow* + 668) and less pseudogenes (*Poc* -93, *Pow* -249). Whilst the number of non-coding genes, including ncRNA, snoRNA, snRNA and tRNA remained comparable. To assess the completeness of the references created, ortholog analysis was performed using the 2 new and 2 historic *P. ovale* spp*.* reference genomes, along with 13 other *Plasmodium* species (see Methods; Supplementary Table S2). A total of 6,916 orthogroups were identified across the 17 *Plasmodium* species references analysed. Of these, 4,268 were identified as being single copy core orthogroups, which were shared across all 13 comparator *Plasmodium* species. Nearly all these orthogroups were represented in Poc221 (99.9%; 4263/4268) and Pow222 (99.9%; 4262/4268), superior to PocGH01 (99.7%; 4255/4268) and PowCR01 (90.1%; 3847/4268), and represent a gain of + 8 and + 415 single copy core orthologs for *Poc* and *Pow,* respectively. This result was subsequently confirmed by BUSCO genome analysis which marked a + 0.3% (*Poc*) and + 0.4% (*Pow*) improvement (Table 2). From the missing single copy core orthogroups, 4 were not identified in any *P. ovale* spp. (Supplementary Table S3), including orthogroup OG0004883, associated with a putative AP2 transcription factor involved in regulating the *Plasmodium* life cycle. Only one single copy core orthogroup (OG0004738; PF3D7_0416500) was present in *Pow* but absent in *Poc,* and vice versa, two were present in *Poc* but not *Pow* (OG0004794; PF3D7_0934500, OG0004804; PF3D7_1460700). The single copy orthologs present in the new *P. ovale* spp. references and the 13 other *Plasmodium* species were used to investigate the phylogeny of *P. ovale* spp*.* The estimated tree topology was in line with previous reports (Supplementary Figure S3), with both *Poc* and *Pow* clustering together. The *P. ovale* spp. clade shares a most recent common ancestor with rodent infecting *Plasmodium* species, including *P. berghei ANKA, P. chabaudi chabaudi, P. vinckei brucechwatti, P. vinckei lentum, P. vinckei vinckei,* and *P. yoelii yoelii*.

**Table 2:** Genome annotation for *P. ovale* spp. references.

Species	*P. ovale curtisi (Poc)*		*P. ovale walker (Pow)*	
Reference	Poc221 (S. Sudan)	PocGH01 (Ghana)	Pow222 (Nigeria)	PowCR01 (Ghana)
Genes	7557	6811	7182	6499
Protein Coding	7440	6671	7063	6395
Non-coding	117	140	119	104
tRNA	68	68	75	58
rRNA	10	37	11	17
snRNA	5	5	5	6
snoRNA	23	21	21	23
ncRNA	11	9	7	0
Pseudogenes	407	500	479	728
Multi-gene families				
PIR genes	1955	1493	1606	1338
STP1 genes	88	67	97	100
BUSCO Completeness (%)	96.2	95.9	95.7	95.3

Due to improvements in genome contiguity, there were additional ortholog chromosomal assignments for both Poc221 (+ 256) and Pow222 (+ 478) compared to the historic *Poc* and *Pow* reference genomes (Supplementary Table S4). Complete mitochondrial sequences were obtained for both Poc221 (5974 bp) and Pow222 (5975 bp), representing almost a 400 bp improvement when compared to the PowCR01 mitochondrial reference (5584 bp). In addition, there were improvements in apicoplast contiguity (3 Kbp added, compared to GenBank entries KX611805 and LT594519), ensuring all 30 core orthogroups were represented (Supplementary Table S5). This result marked an improvement for the *Pow* reference genome with a gain of 11 apicoplast-associated core orthologs.

### *Poc* and *Pow* divergence

**Figure 2:**
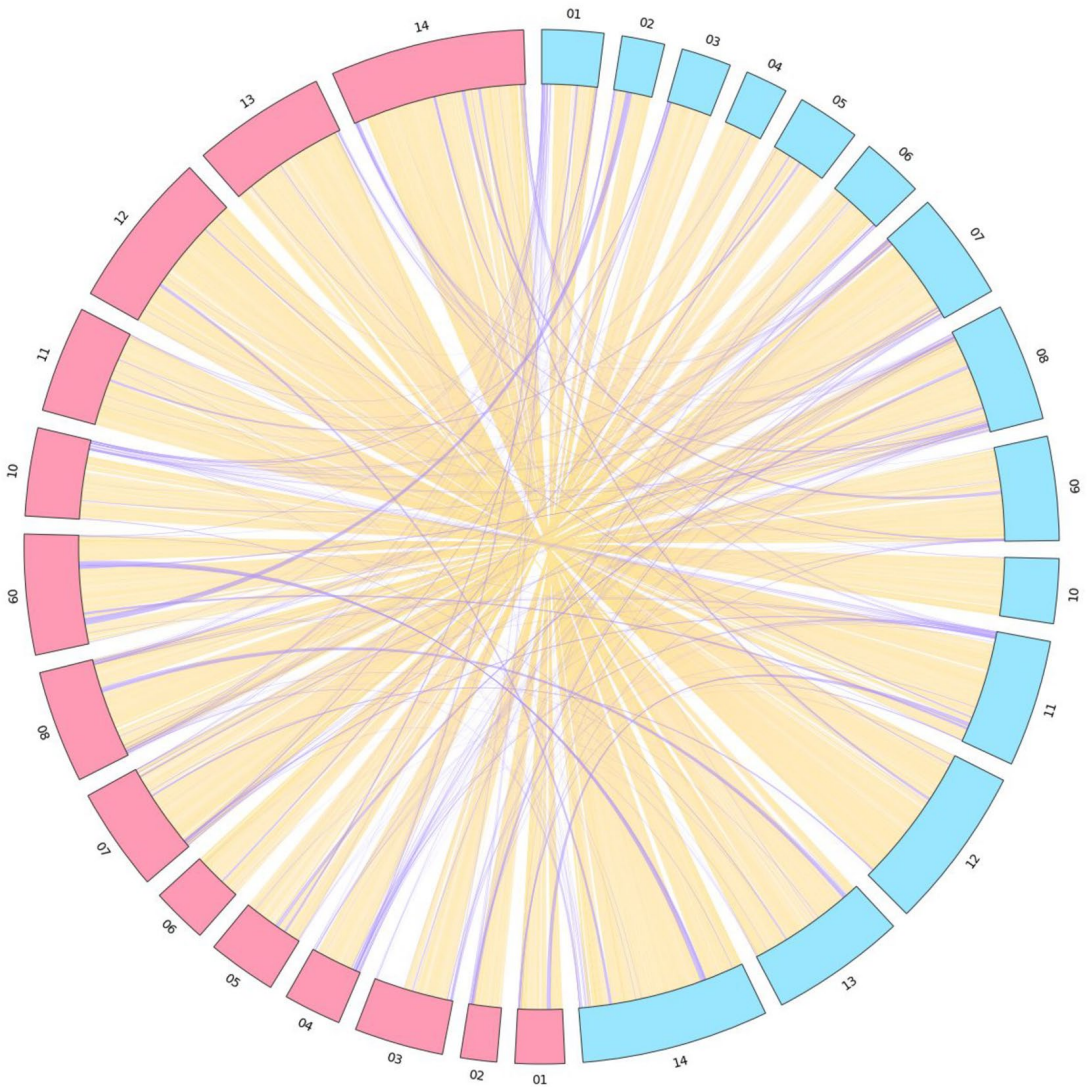
Genomic Islands of homology identified between Poc221 (blue) and Pow222 (red) nuclear chromosomes. Regions located on equivalent chromosomes are shaded Orange, whilst translocated regions above 1kbp are shaded purple.

Expansion of multigene families in *P. ovale* spp*. r*elative to other *Plasmodium* species is a major driver behind genome change^23,24^. The *Plasmodium* interspersed repeat (PIR) multi-gene family is known to be the largest multigene family in most *Plasmodium* species^25^. Additional PIR genes were characterized for both Poc221 (1955 vs. PocGH01 1493) and Pow222 (1606 vs. PowCR01 1338) (Table 2). Of the PIR genes identified, 87.5% (1710) and 95.7% (1429) were clustered into 596 shared orthogroups for Poc221 and PocGH01, respectively. Similarly, 89.6% (1439) and 96.9% (1296) of the PIR genes identified for Pow222 and PowCR01, respectively, are clustered across 586 orthogroups. The results reinforced the greater annotated PIR expansion observed in *Poc* compared to *Pow* (PocGH01 vs. PowCR01: + 155; Poc221 vs Pow222: + 349). Most proteins in the expanded surfin-related subtelomeric protein 1 (STP1/SURFIN) multi-gene family in both *P. ovale* spp. (and *P. malariae*) contain a Schizont-infected cell agglutination (SICA) domain, with SICAvars known to have an evolutionary link with SURFIN and *P. vivax* STP1 proteins^24^. There were similar number of *STP1* genes characterized in Pow222 (97) compared to PowCR01 (100), but more in Poc221 (88) compared to PocGH01 (67). When assessing divergence between shared Poc221 and Pow222 genes, single copy orthologs had a high average protein sequence identity (92.7%). When comparing equivalent nuclear chromosomes between Poc221 and Pow222, they average 81.3% genomic homology, including in sub-telomeric regions**.** No large-scale chromosomal rearrangements were identified between *Poc* and *Pow*, with translocated regions mostly found in sub-telomeric or telomeric regions (Fig. 2).

### Variant calling

To assess the integrity of the new reference genomes, whole genome sequence (WGS) data was analysed for 12 *Poc* and 22 *Pow* samples, sourced publicly (n = 20) and from additional DNA samples from UK travellers (n = 14) (Supplementary Table S6). All samples were mapped to their respective existing and new reference genomes, PocGH01 and Poc221 or PowCR01 and Pow222. There was no significant difference in mean coverage or percentage of reads mapped when comparing the LSHTM sequenced and publicly available samples (all *T*-tests *p* > 0.05)^26^*.* When comparing the percentage of mapped reads between new and existing reference genomes, a slight increase was observed for Poc221 compared to PocGH01 (+ 0.03%: ~ 600 reads) but a significant gain was observed when comparing Pow222 to PowCR01 (+ 0.8%, 204,300 reads, *P* < 3 × 10^–6^). Using a set of 4834 common biallelic SNPs across the 34 isolates, a principal component analysis confirmed the expected two distinct clusters representing the *Poc* and *Pow* species (Supplementary Figure S4).

**Figure 3:**
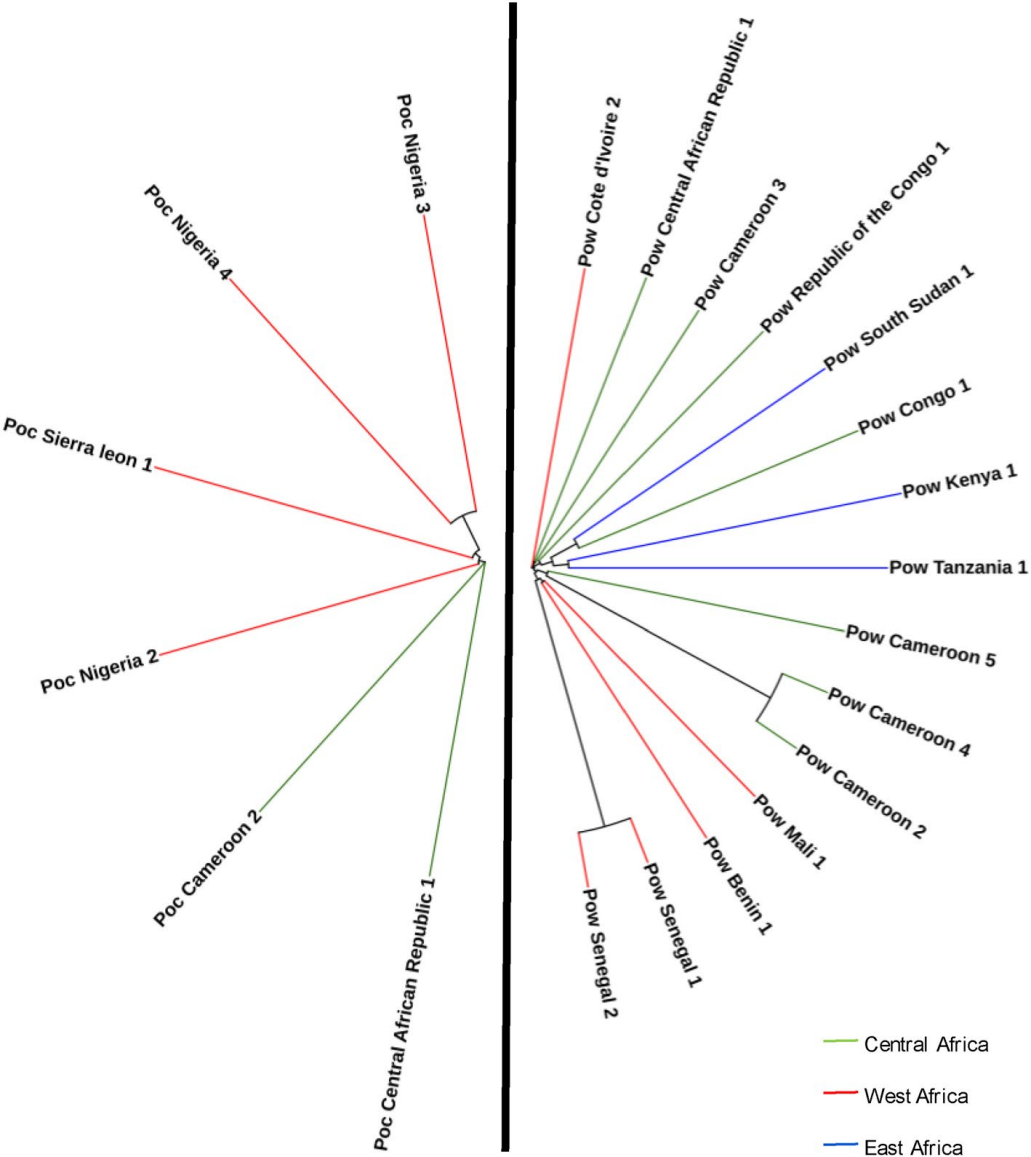
Neighbour joining tree of the 15 *Pow* and 6 *Poc* isolates from West Africa (red), Central Africa (green), East Africa (blue), when aligned to Pow222 and Poc221 respectively. Corresponding BioSample IDs can be found in Supplementary Table S6.

From the 6 *Poc* and 15 *Pow* isolates with high quality genome-wide data, a total of 349,408 and 421,404 genome-wide SNPs were identified, averaging 128,320 and 103,771 SNPs per sample, when utilizing the Poc221 and Pow222 references, respectively. The distribution of nucleotide diversity across both genomes revealed the expected high diversity peaks in sub-telomeric / telomeric regions (Fig. 1), and lower levels in the inferred core genome (Supplementary Table S7). After removing hyper-variable regions, leaving the core genome, 89.3% and 89.0% of the full nuclear genome remained for Poc221 and Pow222, from which a total of 22,517 and 43,855 SNPs were identified from the *Poc* and *Pow* samples, averaging 6,887 and 7,473 SNPs per sample, respectively. Subsequently the nucleotide diversity was estimated (*Poc* 2.98 × 10^–4^; *Pow* 3.43 × 10^–4^), in line with other *Plasmodium* studies^27–29^. Using the SNP data, neighbour-joining trees were constructed for both species, leading to some clustering of *Pow* isolates sourced from Cameroon and Senegal (Fig. 3). Species-specific SNPs in the mitochondrion genomes were confirmed across all isolates, further validating the new reference genomes, and supporting the established paradigm that there is no recombination between *Poc* and *Pow* (Supplementary Table S8).

## Discussion

Regarded as the most recent speciation event within *Plasmodium* malaria parasites, the divergence of the *Poc* and *Pow* genomes is a natural experiment that illuminates the evolutionary forces shaping the adaptive radiation of the genus. To provide insights, we have utilised a combination of paired-end short-read genome sequences with long-read outputs to produce new references genomes for the sibling species *Poc* and *Pow.* These were derived from high-quality material from two clinical isolates harbouring only a single species each, with DNA undergoing further selective whole-genome amplification. Compared to previously available references, which were substantially assembled from co-infecting *P. ovale* spp DNA detected in existing genome sequence data from *P. falciparum-*infected blood samples^23^, we improved overall completeness adding > 400 single copy ortholog gene sequences to the reference annotation of the *Pow* genome. In addition, for both *Poc* and *Pow*, we extended sub-telomeric multi-gene annotations and obtained full organellar genomes for the mitochondrion and apicoplast.

Our robust, two-phase approach provided important new information in three areas. Firstly, more complete nuclear and organellar genome sequence were captured for these species than historic assemblies (PocGH01, PowCR01), with enhanced contiguity. Secondly, telomeric and sub-telomeric hypervariable regions harbouring multigene families such as PIR, believed to play a key role across all life cycle stages of the parasite^25,30^, are comprehensively represented for both species. Thirdly, the long read data identify for the first time a number of chromosomal translocations that are likely to reflect the evolutionary divergence of *Poc* and *Pow* (Fig. 2)*.* This, and the notably greater expansion of the PIR multi-gene family in the genome of *Poc* compared to *Pow*, warrant further investigation and may help us understand the speciation of these two parasites. For example, one open question is whether the varying expansion supports the multi-jump speciation hypothesis, whereby the most common ancestor of *Poc* and *Pow*, was introduced to early homonids via 2 independent host transitions with enough evolutionary time in between to facilitate allopatric speciation by preventing recombination^2^. Further long-read sequencing across many *Poc* and *Pow* isolates, including from non-African sources, could help test whether the observed inter-chromosomal translocations are fixed and potentially contribute to the puzzling lack of recombination between *Poc* and *Pow*, despite their sympatry and the numerous documented co-infections in a single host^31^. Taken together, our data irrefutably support elevation of these two parasite taxa to full species status. This confirms that the *Poc* and *Pow* nomenclature of Sutherland et al*.*^2^, used throughout this report, is incorrect and needs to be changed, as pointed out recently by others^4,5^.

Certain caveats need to be considered in interpreting the data. Clinical ovale malaria infections are invariably of low density, and obtaining samples with sufficient parasite material to generate high quality genome sequences at high coverage is extremely challenging. Our access to additional material to use in our nucleotide diversity analysis was an advantage, but the sample size is very small (only 12 and 22 samples for *Poc* and *Pow*, respectively) and not all produced genome data of the required quality. Further, our *Poc* reference is from East Africa, but ten of our 12 comparators were West African. Conversely, our *Pow* reference is from West Africa, whereas only two isolates of East African origin were available to contribute to the diversity analysis for that species. Therefore, the true pattern of genetic diversity in both *ovale* species can only be determined with broader samples encompassing all of Africa, and parasites from Asia and Oceania. In lieu of such studies, our work provides the first insights into genomic diversity of *Poc* and *Pow*.

With the global objective of malaria eradication, ensuring control and treatment strategies are effective across all human-infecting *Plasmodium* species is essential. The new genome data provided here will improve selection of conserved drug targets for the development of new antimalarials and the development of rapid diagnostic tools which accurately identify these neglected species. These advances are essential to ensure that control activities focusing on *P. falciparum* and *P. vivax* can be modified to also address the burden of morbidity due to ovale malaria.

## Methods

### *P. ovale spp*. samples and DNA processing

Sixteen *P. ovale* spp. DNA samples were extracted from blood samples from returning travellers to the UK, who were diagnosed with malaria between 2019 and 2020, confirmed by the UKHSA MRL at LSHTM. Samples were initially designated as *P. ovale* spp. infections by nested PCR and qPCR according to standard practice. The UK National Research Ethics Service (Ref: 18/LO/0738) and LSHTM Research Ethics Committee (Ref: 14710) provided approval for the whole study under “Drug susceptibility and genetic diversity of imported malaria parasites from UK travellers”, and all methods were performed in accordance with relevant guidelines and regulations, and informed consent was obtained from all UK study participants.

To enrich *Plasmodium* DNA, a *P. ovale* spp*.* selective whole genome amplification (SWGA) primer set was designed, utilizing a software tool (https://github.com/eclarke/swga)^32^, to preferentially amplify *Poc*(PocGH01) and *Pow* (PowCR01) over the human genome (GRCh38). The top outputs were identified and overlapping primers combined to form a final set of 7 primers: CGAAAAA*A*C, CGAAAT*T*G, TCGTAAA*A*A, CGTAAT*A*A, TTTACGT*A*T, ATTTTCG*A*T, and TATCGT*T*A, where an asterisk (*) represents the presence of a phosphorothioate bond which minimises primer degradation by the 3’ exonuclease activity of Phi29. When combined, the SWGA primer set has a total of 28.12 and 28.43 binding sites per 100kbp of PocGH01 and PowCR01, respectively (Supplementary Table S9). There is a total of 2 bindings sites per 100kbp for the GRCh38 human reference genome, indicating a > 14-fold preference for the *Plasmodium* target. Samples were subject to SWGA following previously published protocols^33^. All SWGA reactions were carried out in a UV Cabinet for PCR Operations (UV-B-AR, Grant-Bio) to eliminate potential contamination. A maximum of 80 ng of gDNA (minimum of 5 ng) was added to a total 50 µl reaction alongside 5 µl of 10 × Phi29 DNA Polymerase Reaction Buffer (New England BioLabs), 0.5 µl of Purified 100 × BSA (New England BioLabs), 0.5 µl of 250 µM Primer mix, 5 µl 10 mM dNTP (Roche), 30 units Phi29 DNA Polymerase (New England BioLabs) and Nuclease-Free Water (Ambion, The RNA Company) to reach a final reaction volume of 50 µl. The reaction was carried out on a thermocycler with the following step-down program: 5 min at 35 °C, 10 min at 34 °C, 15 min at 33 °C, 20 min at 32 °C, 25 min 31 °C, 16 h at 30 °C and 10 min at 65 °C. After SWGA, samples were purified using a 1:1 ratio of AMPure XP beads (Beckman-Coulter), following manufacturer’s instructions and quantified via Qubit assay.

### Library preparation and whole genome sequencing (WGS)

Short read sequencing (paired end 150 bp reads) of the DNA samples (n = 16; NCBI accession: PRJNA1015456) was performed on an Illumina NovaSeq 6000 platform by The Applied Genome Centre, LSHTM. For the two isolates (Poc221; SAMN37357391; Pow222 SAMN37357402), selected to create the new reference genomes, long-read sequencing data was obtained in two rounds using ONT MinION made available by The Applied Genome Centre, LSHTM. The two isolates were first prepared for sequencing using the ONT LSK-109 and EXP-NBD104 barcoding kit as per manufacturer's instruction. To select for fragments of greater mass during the library preparation procedure, LSB buffer was used during magnetic bead clean-up and 120 ng of library was loaded onto the R10 flow cell for sequencing with adaptive sampling rejecting reads associated with the human genome (GRCh38). Following SWGA enrichment and T7 endonuclease (NEB-M0302S) treatment, as per manufacturer’s protocol (WAL_9070_v109_revQ_14Aug2019), samples were again prepared following same methodology and sequenced using a R10 flow cell. All resulting fast5 files were base-called using Bonito (ONT) (models: dna_r10.3 and dna_r10.4) and reads generated subsequently trimmed and demultiplexed using Porechop software (v0.2.4). The ONT reads generated had mean read lengths (quality score) of 1.15 Kbp (Q18.2) and 1.17 Kbp (Q18.8) for Poc221 and Pow222 isolates, respectively. All sample specific long-read data obtained was subsequently combined for de novo assembly. In addition, WGS for 20 publicly available *P. ovale* spp. samples were incorporated in this study (ENA project accession: PRJEB51041).

### WGS data quality control

All short read data was filtered via Trimmomatic software (v0.39; parameters: LEADING:3 TRAILING:3 SLIDINGWINDOW:4:20 MINLEN:36)^34^. Long read data was self-mapped via minimap2 (v2.17-r941; parameters: -x ava-ont -g 500)^35^, and scrubbed via *yacrd* (v0.6.2; parameters: default)^36^. Subsequently all sequence data was filtered via Kraken2 (v2.0.7-beta; parameters: default)^37^ and KrakenTools (v1.2; parameters; –exclude –taxid 2 2157 10239 9443 –include-children)^38^, to remove all bacteria, archaea, virus, and primate classified reads. Subsequently all filtered data was mapped to the human reference genome (GRCh38), utilizing minimap2 (v2.17-r941; parameters: -ax map-ont) for long read data and bwa mem (0.7.17-r1188; parameters: default). Following mapping, the samtools software suite was used to extract all reads which were not mapped in a proper pair (v;1.16.1 parameters: view -F 2)^39,40^. Genotypes at biallelic SNPs common to *Poc* and *Pow* were called using GATK, and a principal component analysis was performed to confirm the separation of each species.

### Hybrid genome assembly and annotation

Each stage of the hybrid assembled pipeline used to create the two new references is summarised (Supplementary Figure S1). Initial assemblies were created using Spades software (v3.13.0) under hybrid settings^41^. Contigs was subsequently extended via NTLink (v1.3.8)^42^ and SSPACE (v2.0)^43^ software followed by Pilon (v1.24)^44^ and sniffles (v2.0.7) based polishing^45^. The contigs were then scaffolded via RagTag (v2.1.0)^46^ using the PocGH01 reference as a guide. Gaps in the assembled scaffolds were subsequently closed via Abyss (v2.0.2)^47^, TGS-GapCloser (v1.1.1)^48^ and GapFiller (v1.11)^49^ software tools. Mitochondrial and Apicoplast associated scaffolds were circularized via Circlator (v1.5.5) software^50^, followed by another round of Pilon-based polishing. Each assembly then went through an initial round of annotation via Companion^51^ under default parameters with the prior PocGH01 annotation as a guide. Core orthologs were identified OrthoFinder (v2.5.4)^52^, relative to other *Plasmodium* species. The assemblies were subsequently manually filtered to remove duplicate and misassembled genes based on annotation and coverage metrics. Following manual filtering, scaffolds were split into contigs based on gapped positions and all stages from RagTag (v2.1.0) (43) onwards were rerun. The final assemblies were annotated using both Companion and MetaEUK software (v5.34c21f2)^53^, again using the existing PocGH01 annotation as a guide under default parameters and subsequently assessed via BUSCO v5.3.0, using plasmodium_odb database (2020-08-05). Reference genome summaries were made using the python package pyCircos (v.1.0.2) and custom python scripts which can be found at (https://github.com/MatthewHiggins2017/HigginsMPovaleReferences).

### Ortholog identification

OrthoFinder software (v2.5.4), was utilised to identify orthogroups between the 4 *P. ovale* references (Poc221, Pow222, PocGH01 (PlasmoDB: v54), PowCR01 (NCBI: GCA_900090025.2)) and 13 other *Plasmodium* species; *P. berghei ANKA*, *P. chabaudi chabaudi, P. cynomolgi M, P. falciparum 3D7, P. gallinaceum 8A, P. knowlesi H, P. malariae UG01, P. reichenowi CDC, P. vinckei brucechwatti DA, P. vinckei lentum DE, P. vinckei vinckei CY, P. vivax P01,* and *P. yoelii yoelii 17X*. PIR and STP1 multigene family members were extracted directly from the general feature format (GFF) file for each *P. ovale* spp. reference.

### Phylogenetic analysis

Single copy core orthologroups represented in Poc221 and Pow222 were subsequently extracted and aligned using Mafft software^54^ under default settings. Each alignment was then processed using the GBlocks software^55^ under default settings to remove gapped and uninformative positions. All alignments were subsequently combined to form a sequence covering 1,850,442 amino acids. This combined sequence was used to construct multiple maximum likelihood phylogenetic trees via bootstrapping with RAXML-ng software^56^ utilizing LG substitution model a with gamma distribution. A conserved tree structure for was identified and subsequently visualized via ITOL^57^.

### Genomic Islands of homology

Genomic islands of homology between the new and existing references were identified using progressive Mauve (v2.4.0) software^58^. The program was run with default “seed families” and default values for all other parameters. The same parameters were also used in identified islands of homology between the new Poc221 and Pow222 references.

## Reference validation mapping and variant calling

All Illumina raw sequencing data was filtered using trimmomatic software (v0.39; parameters: LEADING:3 TRAILING:3 SLIDINGWINDOW:4:20 MINLEN:36). Filtered reads were then mapped to the respective new and existing reference genome (Poc221 and PocGH01 or Pow222 and PowCR01) using BWA-MEM alignment (v0.7.17) software. Mapping and coverage statistics were extracted using BWA STATs (v0.7.17) and Samtools (v1.16.1) respectively. SNPs and insertions/deletions (indels) were found using GATK’s HaplotypeCaller (v4.1.4.1)^59^, and subsequently filtering was performed using the GATK VariantFiltration parameters (filter "QD < 2.0" "QUAL < 30.0" "SOR > 3.0" "FS > 60.0" "MQ < 40.0" -"MQRankSum < -12.5" "ReadPosRankSum < -8.0″). SNP density was calculated using vcftools (v0.1.15)^60^. Statistical tests to compare mapping metrics were performed using the Scipy python package^61^. Species-specific SNPs in the mitochondrial *cytB* gene were extracted, and based on using > 90% of sites in a sample, the isolates were correctly classified into *Poc* and *Pow*.

### Supplementary Information

Supplementary Information.

## Data Availability

All raw sequence data is available from NCBI (project accession number PRJNA1015456). All sample accession codes are lists in Supplementary Table S6. The new reference genomes and all associated files can be found at https://github.com/MatthewHiggins2017/HigginsMPovaleReferences/.
