# Supplementary material for: New reference genomes to distinguish the sympatric malaria parasites, Plasmodium ovale curtisi and Plasmodium ovale wallikeri
Source: Sci Rep. 2024 Feb 15;14:3843. doi: 10.1038/s41598-024-54382-5 (PMC10869833; doi:10.1038/s41598-024-54382-5)

**Supplementary Information**

**New reference genomes to distinguish the sympatric malaria parasites, *Plasmodium ovale curtisi* and *Plasmodium ovale wallikeri***

Matthew Higgins ^1^, Emilia Manko ^1^, Daniel Ward ^1^, Jody E. Phelan ^1^**,** Debbie Nolder ^1,2^, Colin J. Sutherland ^1,2^, Taane G. Clark ^1,3,*^, Susana Campino ^1,*,^

1. Faculty of Infectious and Tropical Diseases, London School of Hygiene & Tropical Medicine, WC1E 7HT London, UK
2. UK Health Security Agency, Malaria Reference Laboratory, London School of Hygiene & Tropical Medicine, Keppel Street, London WC1E 7HT, United Kingdom
3. Faculty of Epidemiology and Population Health, London School of Hygiene & Tropical Medicine, WC1E 7HT London, UK

* Joint corresponding authors

Supplementary Tables

**Supplementary Table 1.** Summary of whole genome sequencing (WGS) yield (Gbp) for Poc221 and Pow222 through the analysis pipeline (see **Methods**).

|  |  | WGS Data Present (Gbp) | | | | |
| --- | --- | --- | --- | --- | --- | --- |
| Sample | Platform | Original | Trimming | Scrubbing | Kraken software Decontamination | Mapping Decontamination |
| Poc221 | Illumina | 11.58 | 10.56 | - | 4.04 | 2.40 |
| Poc221 | ONT | 2.10 | - | 1.20 | 0.64 | 0.56 |
| Pow222 | Illumina | 10.32 | 9.42 | - | 3.44 | 1.68 |
| Pow222 | ONT | 3.46 | - | 1.42 | 0.83 | 0.72 |

ONT Oxford Nanopore Technology

**Supplementary Table 2.** Ortholog Analysis. A total of 6,916 ortholog groups identified when analysing 17 *Plasmodium r*eferences incorporating 13 non-ovale species, 2 *P. ovale curtisi (Poc)* and 2 *P. ovale wallikeri (Pow) references*. Shared Orthologs are all groups which contain at least one gene for any of the 13 non-ovale *Plasmodium* species. Core Orthologs are all groups which contain at least one gene from all 13 non-ovale *Plasmodium* species. Single Copy Orthologs, contains only one gene from all 13 non-ovale *Plasmodium* species.

|  | **Shared Orthogroups** | **Core Orthogroups** | **Single Copy Core Orthogroups** | | |
| --- | --- | --- | --- | --- | --- |
|  |  |  | **Single** | **Duplicated** | **Missing** |
| **Total** | 5784 | 4404 | 4268 | | |
| **Poc221** | 4957 | 4398 | 4255 | 8 | 5 |
| **PocGH01** | 4911 | 4388 | 4253 | 2 | 13 |
| **Pow222** | 4952 | 4397 | 4251 | 11 | 6 |
| **PowCR01** | 4436 | 3967 | 3840 | 7 | 421 |

**Supplementary Table 3.** Single copy core orthogroups missing across all *P. ovale* spp references.

| **Orthogroup** | ***P. falciparum* ortholog** | **Ortholog Description** |
| --- | --- | --- |
| **OG0004878** | *PF3D7_0218200* | SUZ domain-containing protein, unknown function. |
| **OG0004879** | *PF3D7_0421900* | Conserved *Plasmodium* protein, unknown function. |
| **OG0004882** | *PF3D7_1120700* | Conserved *Plasmodium* protein, unknown function. |
| **OG0004883** | *PF3D7_0404100* | AP2 domain transcription factor AP2-SP2, putative. |

**Supplementary Table 4.** Comparison of 1:1 ortholog chromosomal assignment between historic assemblies and new reference genomes (Poc221 versus PocGH01 and Pow222 versus PowCR01)

| **Species** | ***Poc*** | ***Pow*** |
| --- | --- | --- |
| 1:1 Orthologs | 5249 | 4085 |
| Assigned to Chromosome Both | 4689 | 4804 |
| Assigned to Chromosome New Only | 270 | 508 |
| Assigned to Chromosome Old Only | 14 | 30 |
| Not Assigned | 276 | 181 |
| **Net Change** | **+256** | **+478** |

*P. ovale curtisi* (*Poc*), *P. ovale wallikeri* (*Pow)*

**Supplementary Table 5**. Apicoplast core orthogroups, represented in *P. ovale* spp. references and *P. falciparum 3D7*. - indicates the ortholog is missing in the given reference.

| **Orthogroup** | **Pf3D7** | **PowCR01** | **PocGH01** | **Poc221** | **Pow222** |
| --- | --- | --- | --- | --- | --- |
| OG0003652 | API00100 | SBT7906 | API000200 | API0005000 | API0005000 |
| OG0003653 | API01300 | SBT7906 | API001200 | API0005800 | API0006000 |
| OG0004848 | API01400 | - | API001300 | API0009800 | API0010400 |
| OG0003654 | API01500 | SBT79065 | API001400 | API0005900 | API0006100 |
| OG0003655 | API01600 | SBT7906 | API001500 | API0006000 | API0006200 |
| OG0004719 | API01700 | - | API001600 | API0006100 | API0006300 |
| OG0004718 | API01800 | - | API001700 | API0006200 | API0006400 |
| OG0004847 | API01900 | - | API001800 | API0009900 | API0010500 |
| OG0003656 | API02000 | SBT7906 | API001900 | API0006300 | API0006500 |
| OG0004774 | API02100 | SBT7906 | API002000 | API0006400 | API0006600 |
| OG0003657 | API02200 | SBT7907 | API002100 | API0010000 | API0010600 |
| OG0004717 | API02300 | - | API002200 | API0006500 | API0006700 |
| OG0004832 | API02400 | SBT7907 | API002300 | API0006600 | API0006800 |
| OG0004846 | API02500 | - | API002400 | API0010100 | API0010700 |
| OG0004716 | API02600 | - | API002500 | API0006700 | API0006900 |
| OG0003658 | API02700 | SBT7907 | API002600 | API0006800 | API0007000 |
| OG0003659 | API02800 | SBT7907 | API002700 | API0006900 | API0007100 |
| OG0003660 | API02900 | SBT7907 | API002800 | API0007000 | API0007200 |
| OG0003661 | API03000 | SBT7907 | API002900 | API0007100 | API0007300 |
| OG0003662 | API03500 | SBT7907 | API003400 | API0007600 | API0007800 |
| OG0003663 | API03600 | SBT7907 | API003500 | API0007700 | API0007900 |
| OG0004715 | API03800 | - | API003700 | API0007900 | API0008100 |
| OG0004845 | API04000 | - | API003900 | API0010200 | API0010800 |
| OG0003664 | API04100 | SBT7908 | API004000 | API0008100 | API0008300 |
| OG0004714 | API04200 | - | API004100 | API0008200 | API0008400 |
| OG0003665 | API04300 | SBT7908 | API004200 | API0010300 | API0008500 |
| OG0004713 | API04400 | - | API004300 | API0008300 | API0008600 |
| OG0003666 | API04500 | SBT7908 | API004400 | API0008400 | API0008700 |
| OG0003667 | API04600 | SBT7908 | API004500 | API0008500 | API0008800 |
| OG0003668 | API04700 | SBT7908 | API004600 | API0008600 | API0008900 |

**Supplementary Table 6**. Metadata for the new reference genomes (Poc221, Pow222) and 34 *P. ovale* spp. samples used to assess them.

| **BioSample** | **Speciation** | **Location** | **Figure ID** |
| --- | --- | --- | --- |
| SAMEA13060846 | Poc | Cameroon | Poc Cameroon 1 |
| SAMN37357394 | Poc | Cameroon | Poc Cameroon 2 |
| SAMEA13060845 | Poc | Central African Republic | Poc Central African Republic 1 |
| SAMEA13060844 | Poc | Gabon | Poc Gabon 1 |
| SAMN37357389 | Poc | Kenya | Poc Kenya 1 |
| SAMN37357388 | Poc | Nigeria | Poc Nigeria 1 |
| SAMN37357392 | Poc | Nigeria | Poc Nigeria 2 |
| SAMN37357393 | Poc | Nigeria | Poc Nigeria 3 |
| SAMN37357395 | Poc | Nigeria | Poc Nigeria 4 |
| SAMEA13060843 | Poc | Republic of the Congo | Poc Republic of the Congo 1 |
| SAMN37357396 | Poc | Sierra Leone | Poc Sierra Leone 1 |
| SAMN37357391 (*Poc221*) | Poc | South Sudan | Poc South Sudan 1 |
| SAMN37357390 | Poc | Uganda | Poc Uganda 1 |
| SAMEA13060850 | Pow | Benin | Pow Benin 1 |
| SAMEA112281809 | Pow | Cameroon | Pow Cameroon 1 |
| SAMEA112281800 | Pow | Cameroon | Pow Cameroon 2 |
| SAMEA112281807 | Pow | Cameroon | Pow Cameroon 3 |
| SAMEA13060848 | Pow | Cameroon | Pow Cameroon 4 |
| SAMEA13060851 | Pow | Cameroon | Pow Cameroon 5 |
| SAMEA112281805 | Pow | Cameroon | Pow Cameroon 6 |
| SAMEA112281811 | Pow | Cameroon | Pow Cameroon 7 |
| SAMEA13060852 | Pow | Central African Republic | Pow Central African Republic 1 |
| SAMN37357403 | Pow | Congo | Pow Congo 1 |
| SAMEA112281808 | Pow | Cote d'Ivoire | Pow Cote d'Ivoire 1 |
| SAMEA112281803 | Pow | Cote d'Ivoire | Pow Cote d'Ivoire 2 |
| SAMEA112281804 | Pow | Cote d'Ivoire | Pow Cote d'Ivoire 3 |
| SAMN37357400 | Pow | Kenya | Pow Kenya 1 |
| SAMEA13060849 | Pow | Mali | Pow Mali 1 |
| SAMN37357402 (*Pow222*) | Pow | Nigeria | Pow Nigeria 1 |
| SAMEA13060853 | Pow | Republic of the Congo | Pow Republic of the Congo 1 |
| SAMEA112281810 | Pow | Senegal | Pow Senegal 1 |
| SAMEA112281806 | Pow | Senegal | Pow Senegal 2 |
| SAMN37357401 | Pow | South Sudan | Pow South Sudan 1 |
| SAMN37357399 | Pow | Tanzania | Pow Tanzania 1 |
| SAMN37357397 | Pow | Uganda | Pow Uganda 1 |
| SAMN37357398 | Pow | Zambia | Pow Zambia 1 |

*P. ovale curtisi* (*Poc*), *P. ovale wallikeri* (*Pow)*

**Supplementary Table 7.** Core genome coordinates, excluding hyper-variable regions.

| **Species** | **Chromo** | **Length** | **Core Start** | **Core End** | **Core Size** |
| --- | --- | --- | --- | --- | --- |
| *P. ovale curtisi* (Poc221) | 1 | 1159247 | 190000 | 1080000 | 890000 |
|  | 2 | 805468 | 0 | 750000 | 750000 |
|  | 3 | 939301 | 130000 | 930000 | 800000 |
|  | 4 | 799619 | 0 | 799619 | 799619 |
|  | 5 | 1183063 | 10000 | 1183063 | 1173063 |
|  | 6 | 1165930 | 0 | 950000 | 950000 |
|  | 7 | 2030079 | 290000 | 1770000 | 1480000 |
|  | 8 | 2214511 | 260000 | 1990000 | 1730000 |
|  | 9 | 1951678 | 170000 | 1880000 | 1710000 |
|  | 10 | 1205626 | 0 | 1030000 | 1030000 |
|  | 11 | 2384891 | 160000 | 2384891 | 2224891 |
|  | 12 | 3064923 | 0 | 3040000 | 3040000 |
|  | 13 | 2537708 | 90000 | 2537708 | 2447708 |
|  | 14 | 3516496 | 0 | 3260000 | 3260000 |
| *P. ovale wallikeri* (Pow222) | 1 | 928759 | 0 | 910000 | 910000 |
|  | 2 | 681398 | 0 | 570000 | 570000 |
|  | 3 | 1673684 | 120000 | 1060000 | 940000 |
|  | 4 | 1086182 | 170000 | 1070000 | 900000 |
|  | 5 | 1291776 | 40000 | 1291776 | 1251776 |
|  | 6 | 1055110 | 0 | 920000 | 920000 |
|  | 7 | 1941600 | 120000 | 1760000 | 1640000 |
|  | 8 | 2115125 | 110000 | 2010000 | 1900000 |
|  | 9 | 2253887 | 200000 | 2190000 | 1990000 |
|  | 10 | 1653700 | 0 | 1110000 | 1110000 |
|  | 11 | 2005720 | 0 | 1990000 | 1990000 |
|  | 12 | 2979699 | 0 | 2930000 | 2930000 |
|  | 13 | 2509376 | 80000 | 2430000 | 2350000 |
|  | 14 | 3645926 | 40000 | 3610000 | 3570000 |

**Supplementary Table 8.** C*ytB* mitochondrial barcoding SNPs (haplotype) for *P. ovale curtisi* (*Poc*) and *P. ovale wallikeri* (*Pow)*, within the existing PocGH01 reference.

| PocGH01  Position* | Nucleotide | |
| --- | --- | --- |
|  | *Poc* | *Pow* |
| 1114 | T | G |
| 1075 | T | C |
| 901 | A | T |
| 874 | G | A |
| 784 | A | T |
| 742 | G | A |
| 532 | C | A |
| 520 | G | A |
| 502 | A | T |
| 391 | G | A |
| 373 | G | A |
| 328 | A | T |

* based on PocGH01_MIT_v2;

**Supplementary Table 9**. The number of binding sites for each SWGA primer across each *P. ovale* spp. reference genome and expected human background. A comparison against Joste *et al.* 2022 *P. ovale* SWGA primer sets (DOI: 10.1128/spectrum.00726-22) is presented.

|  | ***P. ovale curtisi*** | | ***P. ovale wallikeri*** | | ***Human*** |
| --- | --- | --- | --- | --- | --- |
|  | **Poc221** | **PocGH01** | **Pow222** | **PowCR01** | **GRCh38** |
| ***P. ovale* spp.** |  |  |  |  |  |
| ATTTTCGAT | 763 | 686 | 737 | 703 | 4969 |
| CGAAATTG | 1746 | 1646 | 1783 | 1758 | 12837 |
| TATCGTTA | 1796 | 1701 | 1723 | 1658 | 14757 |
| CGAAAAAAC | 655 | 626 | 654 | 643 | 4109 |
| TCGTAAAAA | 1508 | 1432 | 1515 | 1461 | 5163 |
| TTTACGTAT | 1431 | 1321 | 1343 | 1296 | 9887 |
| CGTAATAA | 2261 | 2005 | 2079 | 2013 | 16061 |
| **Total Binding Sites Per 100kbp** | **28.17** | **28.12** | **28.59** | **28.43** | **2.06** |
| **Joste et al. *Poc*** |  |  |  |  |  |
| ATATTTTCG | 1253 | 1174 | - | - | 6931 |
| CGTATCG | 921 | 865 | - | - | 5347 |
| TAATTCGTA | 833 | 777 | - | - | 3590 |
| TATTTCGTA | 1221 | 1122 | - | - | 5449 |
| TCGTATATA | 1166 | 1044 | - | - | 4905 |
| **Total Binding Sites Per 100kbp** | **14.95** | **14.88** | - | - | **0.79** |
| **Joste *et al. Pow*** |  |  |  |  |  |
| ATATACGAA | - | - | 1383 | 1360 | 6184 |
| CGATAAAAA | - | - | 1085 | 1064 | 7280 |
| CGATACG | - | - | 993 | 976 | 5347 |
| TACGAAATA | - | - | 1128 | 1083 | 5449 |
| TATAACGAA | - | - | 950 | 910 | 4830 |
| **Total Binding Sites Per 100kbp** | - | - | **16.10** | **16.08** | **0.88** |

Supplementary Figures

**Supplementary Figure 1.** Summary of the hybrid assembly pipeline used for *Poc221* and *Pow222* reference assembly.


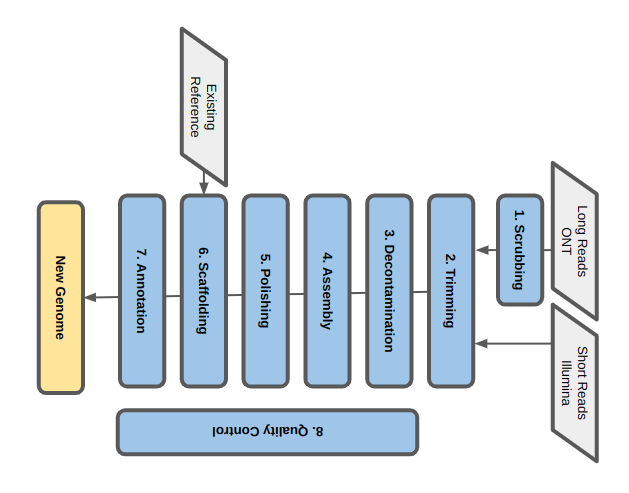


**Supplementary Figure 2.** Gains in contiguity across nuclear chromosomes when benchmarking.

**A)** Poc221 vs. PocGH01


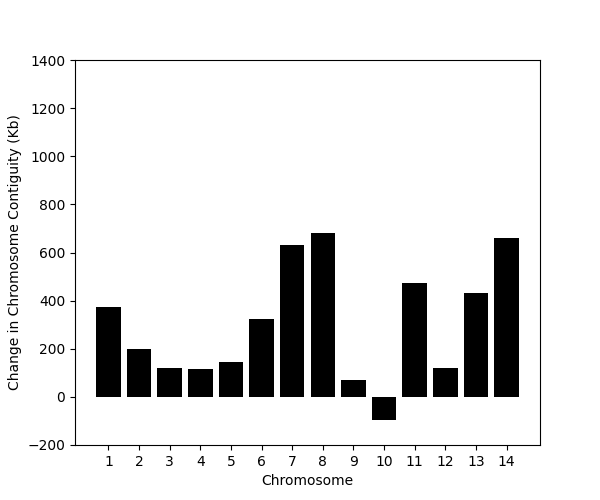


**B)** Pow222 vs. PowCR01


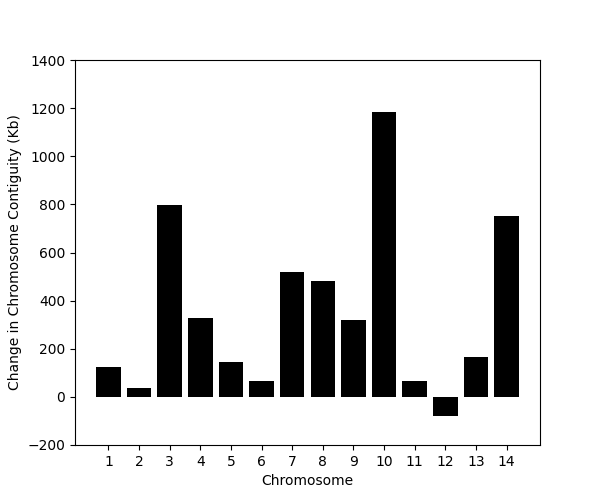


**Supplementary Figure 3.** Phylogenetic placement of *P. ovale* spp*.* when using the new references Poc221 and Pow222, generated using RAXML-NG with a LG+G evolutionary model.


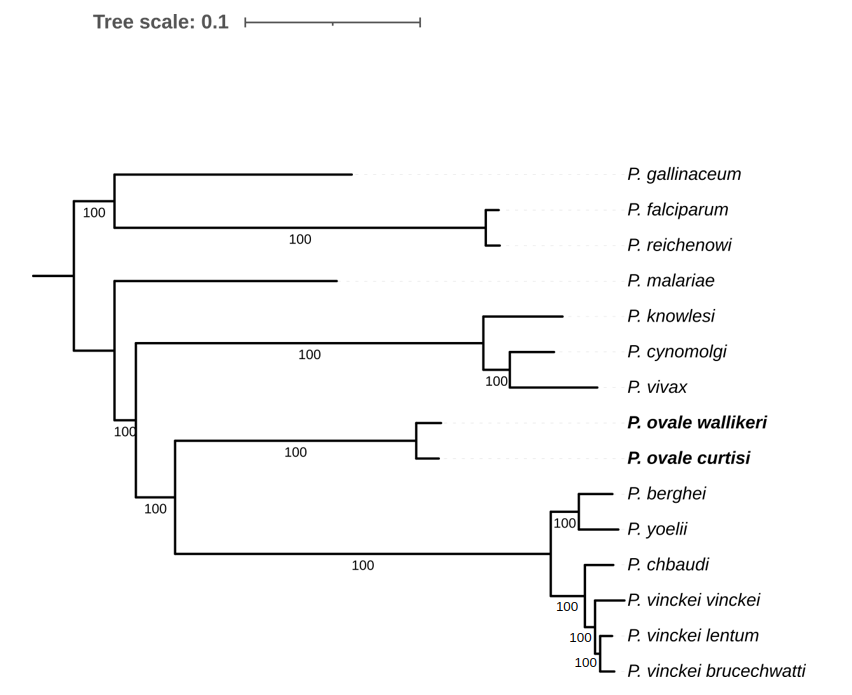


**Supplementary Figure 4.** Principal Component Analysis of all *P. ovale curtisi* (*Poc*; Blue; n=12) and *P. ovale wallikeri* (*Pow;* Red; n=22) speciated isolates.


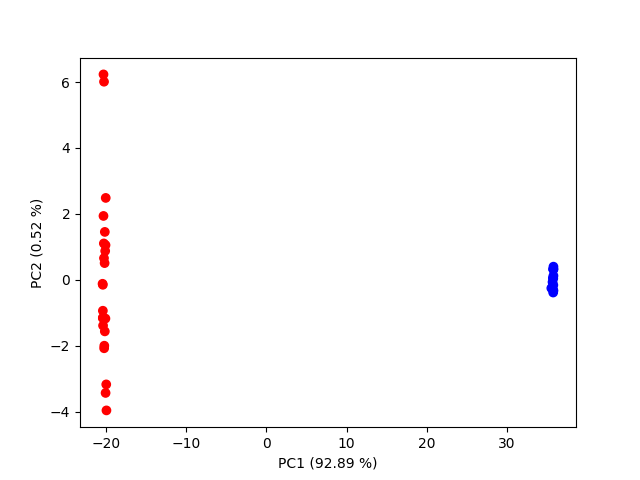

Supplement: Supplementary file 1 — Supplementary Information. [file 41598_2024_54382_MOESM1_ESM.docx]
